# Cerebrovascular Function in Hormonal Migraine: An Exploratory Study

**DOI:** 10.3389/fneur.2021.694980

**Published:** 2021-07-07

**Authors:** Jemima S. A. Dzator, Peter R. C. Howe, Lyn R. Griffiths, Kirsten G. Coupland, Rachel H. X. Wong

**Affiliations:** ^1^School of Biomedical Sciences and Pharmacy, University of Newcastle, Callaghan, NSW, Australia; ^2^Adelaide Medical School, University of Adelaide, Adelaide, SA, Australia; ^3^Genomics Research Centre, School of Biomedical Sciences, Centre for Genomics and Personalised Health, Queensland University of Technology, Brisbane, QLD, Australia; ^4^Hunter Medical Research Institute, New Lambton Heights, NSW, Australia; ^5^Centre for Health Research, Institute for Resilient Regions, University of Southern Queensland, Raceview, QLD, Australia

**Keywords:** cerebral blood flow, cerebrovascular function, hormonal migraine, neurovascular coupling, transcranial Doppler

## Abstract

**Background:** Migraineurs, particularly young premenopausal women, are at increased risk of cerebrovascular disease; however, there is currently limited evidence as to whether hormonal migraine is associated with poor cerebrovascular function.

**Objectives:** The objectives of this study were to: (1) investigate the potential association of cerebrovascular function with hormonal migraine and (2) determine whether abnormalities of cerebrovascular function in hormonal migraineurs are associated with migraine-related disability and/or quality of life.

**Method:** A cross-sectional study was undertaken in 50 hormonal migraineurs (mean age: 38.7 ± 1.2 years) and 29 controls (mean age: 35.6 ± 1.8 years). Data were collected at a single point in time from all participants during the inter-ictal period when they were free from migraine and not menstruating. Transcranial Doppler ultrasound was used to measure resting blood flow velocity and cerebrovascular responsiveness (CVR) to hypercapnia and cognitive stimulation (neurovascular coupling) in the left and right middle cerebral artery (MCA). Additionally, hormonal migraineurs completed three questionnaires to assess migraine-related disability and quality of life as well as migraine frequency and intensity: Headache Impact Test-6™, Migraine-Specific Quality of Life and Migraine Disability Assessment.

**Results:** Hormonal migraineurs had lower resting mean blood flow velocity (MBFV) (*P* = 0.009) and neurovascular coupling during cognitive stimulation (*P* = 0.010) in the left MCA than controls. No such differences were found in the right MCA. Additionally, heart rate (*P* = 0.004) was higher in hormonal migraineurs than controls. However, no differences in CVR to hypercapnia were found between hormonal migraineurs and controls. Multi-variate analysis revealed age to be a significant (*P* = 0.012) predictor of MBFV in the left MCA. Negative correlations between headache frequency and CVR to hypercapnia in the left (*P* = 0.026) and right MCA (*P* = 0.044) were found. Additionally, negative correlations between neurovascular coupling during the 2-Back 1.5 s task in the right MCA and the MSQoL emotional (*P* = 0.013) and role-function restrictive (*P* = 0.039) domains were found.

**Conclusions:** This is the first study to show that hormonal migraineurs have poorer cerebrovascular function, as represented by lower resting MBFV and impaired neurovascular coupling in the left MCA. Future studies should investigate whether improving cerebrovascular function can prevent hormonal migraine and improve quality of life.

**Clinical Trial Registration:** ACTRN12618001230246.

## Introduction

Migraine is a common neurovascular disorder characterised by a severe throbbing headache that can last anywhere between 4 and 72 h (1, 2). Estimated to affect 10% of the global population, migraine disproportionately affects females, who are three times more likely to suffer from migraine than males (2, 3). This difference is evident after puberty, and is believed to be influenced by the sex hormones, particularly oestrogen (4).

More than half of female migraineurs have migraine attacks, often without aura, prior to menstruation or during ovulation (5–7). Known as hormonal migraines, these migraines are triggered by the rapid decrease of oestrogen that occurs prior to menstruation (8), or during ovulation (4–6, 9). Oestrogen is vital for the healthy functioning of the vascular endothelium as it reduces inflammation, oxidative stress and cell death and importantly regulates the vasomotor tone of blood vessels (10, 11). Therefore, acute oestrogen withdrawal prior to menstruation may diminish endothelial function, potentially undermining the regulation of vasomotor tone. Hence, it is plausible that hormonal migraineurs may have suboptimal endothelial function and the rapid withdrawal of oestrogen that occurs prior to menstruation or during ovulation may worsen the cerebral endothelial dysfunction and thereby trigger the migraine. Findings from a recent meta-analysis are consistent with this hypothesis (12). However, there is currently a paucity of research linking poor cerebrovascular function to hormonal migraine. Interestingly, migraine is associated with an increased risk of cerebrovascular disease, such as stroke, particularly in young premenopausal women (13–15). Therefore, an association between poorer cerebrovascular function and hormonal migraine may partially account for the increased risk of cerebrovascular disease in young premenopausal women with migraine.

Studies have shown that women who suffer from hormonal migraine, have attacks that are longer in duration, more incapacitating, recurrent, painful and resistant to treatment compared to their non-hormonal migraine counterparts (5, 16). However, few studies have assessed migraine-related quality of life and migraine-related disability in a population of hormonal migraineurs. Furthermore, to our knowledge, no study has assessed whether migraine-related quality of life or migraine-related disability is associated with poorer cerebrovascular function in hormonal migraineurs. Therefore, the main objectives of this study were to investigate whether hormonal migraineurs have poorer cerebrovascular function than healthy controls and determine whether migraine-related quality of life and disability are associated with cerebrovascular function.

## Materials and Methods

### Study Design and Participants

A cross-sectional study was conducted in 50 women with hormonal migraine (mean age: 38.7 ± 1.2 years) and 29 healthy women (mean age: 35.6 ± 1.8 years) at the Clinical Nutrition Research Centre, University of Newcastle, Australia. Participants were recruited from the Hunter Region in New South Wales between August 2018 and December 2019 by advertising *via* newspaper, radio, social media platforms and distributing flyers. Women who expressed interest in participating in the study were instructed to complete an online Menstrual Headache, Health and Lifestyle screening questionnaire to determine their suitability for the study. If deemed suitable, participants were invited to visit the research centre on one occasion to confirm their eligibility and undergo baseline assessments. All participants visited the research centre when they were free from migraine, not menstruating and fasted for at least 1 h prior to the visit. Data were collected during the inter-ictal period for hormonal migraineurs.

Written informed consent was obtained from all participants prior to baseline assessments. This study was approved by the University of Newcastle Human Research Ethics Committee (H-2018-0167) and is registered with the Australian and New Zealand Clinical Trials Registry (ACTRN12618001230246).

### Inclusion Criteria

Eligible participants for both the hormonal migraine and control groups were required to be aged between 18 years and menopause (<6 month since cessation of menses). All participants were required to have a detectable transcranial Doppler (TCD) ultrasound signal in the MCA for the CVR to hypercapnia procedure. Participants in the control group were required to not suffer from any type of migraine. Participants in the hormonal migraine group were required to have migraines with nausea/vomiting, photophobia, phonophobia and/or osmophobia lasting at least 4 h with at least two of the following characteristics: pulsating or throbbing pain, pain of at least moderate intensity, unilateral location, or pain aggravated by or causing avoidance of physical activity. Additionally, participants were required to have migraines in association with (± 3 days from) the onset of their period or during ovulation in the three previous menstrual cycles prior to the visit (6, 7). The incidence of migraine in women increases 3 days prior to the onset of menstruation (17, 18). Therefore, the peri-hormonal period for this study was defined as migraines that occurred within ±3 days from the onset of the participant's period. The migraine symptomatology of participants in the hormonal migraine group was obtained *via* the Menstrual Headache, Health and Lifestyle screening questionnaire and during the baseline/screening visit.

### Exclusion Criteria

Participants were excluded if they were pregnant, breastfeeding, or had any of the following: liver disease, kidney disease, stroke, epilepsy, Chiari malformation, Parkinson's disease, unmanaged major depression, fibromyalgia, insulin-dependent diabetes, hysterectomy, illiteracy or non-hormonal migraine. Participants who were smokers or had a history of alcohol or drug abuse were also excluded. Participants were not excluded if they were taking any migraine medication or hormonal contraception, provided that they were still menstruating and able to track their menstrual cycle.

### Blood Pressure and Arterial Compliance

Blood pressure, heart rate and arterial compliance were simultaneously measured whilst seated using the HDI/PulseWave™ CR2000 Research Cardiovascular Profiling System. Participants were fitted with an appropriately sized blood pressure cuff, positioned over the brachial artery of their non-dominant arm, to measure their blood pressure. A tonometer was positioned over the radial artery of their dominant arm to measure the compliance of their small and large arteries. Four consecutive readings of blood pressure, heart rate and arterial compliance were taken at 5 min intervals with the first reading discarded and the remaining three readings averaged.

### Measurement of Cerebrovascular Function

Transcranial Doppler (TCD) ultrasound (Doppler BoxX, Compumedics DWL, Germany) was used to measure pulsatile blood flow velocity (BFV) bilaterally in the middle cerebral artery (MCA) at rest and under dynamic conditions. Resting maximum, mean and minimum BFV were used to assess intracranial arterial stiffness as follows: Gosling pulsatility index (PI) = (Maximum BFV—Minimum BFV)/Mean BFV; Pourcelot resistive index (RI) = (Maximum BFV—Minimum BFV)/Maximum BFV.

Changes of BFV in the MCA were measured whilst participants inhaled carbogen gas (95% O_2_, 5% CO_2_) for 3 min. Cerebrovascular responsiveness (CVR) to this hypercapnic stimulus was calculated as follows: CVR (%) = (Peak BFV—Basal BFV)/Basal BFV) × 100.

Participants also completed an 8 min N-Back test whilst changes in BFV in the MCA were measured to determine CVR to this cognitive demand (neurovascular coupling). The N-Back test was administered on an iPad. Participants completed three conditions of the N-Back test consecutively with increasing difficulty. The first condition was the 1-Back, in which participants were shown a series of letters that appeared and disappeared on the iPad screen one at a time, for ~1 min. Each letter appeared on the screen for 2 s before a new letter replaced it. Participants were instructed to tap the screen if the current letter displayed on the screen was identical to the letter that was shown one-step back in the sequence. Participants then completed two conditions of a 2-Back test, during which they were instructed to tap the screen if the letter displayed was identical to the letter shown two steps back in the sequence. During the first and second conditions of the 2-Back test, participants had 1.5 and 1.0 s respectively between each letter to decide whether to tap the screen. The 2-Back 1.5 s test and 2-Back 1.0 s test lasted for ~2 and 1 min, respectively.

Table Curve 2D Version 5.01 (SYSTAT Software Incorporated, 2002) was used to determine the basal and peak MBFV of each recording during CVR to hypercapnia and neurovascular coupling. The smooth data spline estimation function with Loess curve fitting was used with 10 and 20% smoothing for neurovascular coupling and CVR to hypercapnia, respectively (19).

### Assessment of Migraine-Related Quality of Life and Disability

Participants from the hormonal migraine group completed three questionnaires that assessed migraine-related disability and quality of life: the Headache Impact Test-6 (HIT-6™) (20), the Migraine Disability Assessment (MIDAS) (21), and the Migraine Specific Quality of Life (MSQoL) (22). The HIT-6™ assesses the impact of headaches on an individual's ability to function at work, home and during social situations. The total HIT-6™ score determines the headache impact severity grade (<49 = little or no impact, 50–55 = some impact, 56–59 = substantial impact, and 60–78 = severe impact) (20). The MIDAS assesses to what extent migraine limits an individual's ability to perform activities. The total MIDAS score determines the grade of migraine-related disability (little or no disability = 0–5, mild disability = 6–10, moderate disability = 11–20, severe disability ≥21). The MIDAS questionnaire has two additional items, MIDAS item A (frequency of migraine during the last three previous months) and MIDAS item B (average intensity of the participant's migraine) (21). The MSQoL questionnaire measures how migraine affects an individual's quality of life across three domains; role-function restrictive (the degree to which migraine limits an individual's ability to undertake daily activities), role-function preventive (the degree to which migraine prevents an individual from undertaking daily activities) and emotional function (the degree to which migraine produces feelings of helpless and frustration). The higher the MSQoL score, the better the self-perceived quality of life (22).

### Sample Size and Statistical Analysis

A total of 34 participants in each group were needed to give 80% power to see a statistically significant (*p* < 0.05) difference of large effect size (Cohen's *d* = 0.7) in the primary outcome (CVR to hypercapnia) between hormonal migraineurs and controls. However, due to challenges in recruitment, only 29 controls were finally enrolled into the study. We sought to offset this deficit by recruiting more hormonal migraine participants (50 enrolled) on the assumption that variance in the primary outcome was likely to be greater in this group.

Statistical analyses were performed using SPSS Version 25.0 for Windows (IBM Corp. Armonk, NY, USA). The normality of the data distribution was assessed using the Anderson-Darling normality test. Student's *t-*test was used for normally distributed data to compare the means of the outcomes between the hormonal migraine and control group. The Wilcoxon-Mann-Whitney test was used for data that was not normally distributed. The associations between measures of cerebrovascular function (i.e., mean BFV, CVR to hypercapnia and neurovascular), and measures of cardiovascular function and migraine-related quality of life, disability, intensity and frequency were investigated using a generalised linear model. Variables that were statistically significant (*p* < 0.1) in univariate analyses were included in the multi-variate model. Univariate and multi-variate linear regression models were performed in both the total study population, and hormonal migraineurs sub-group. Multi-collinearity was assessed using the variance inflation factor (VIF); variables with a VIF of >2.5 were considered to be indicative of multi-collinearity. The significance level for all other results was set at *p* = 0.05 with no adjustment for multiple comparisons as this was an exploratory study. All values are presented as mean ± SEM unless otherwise specified.

## Results

### Participant Characteristics

**Figure 1:**
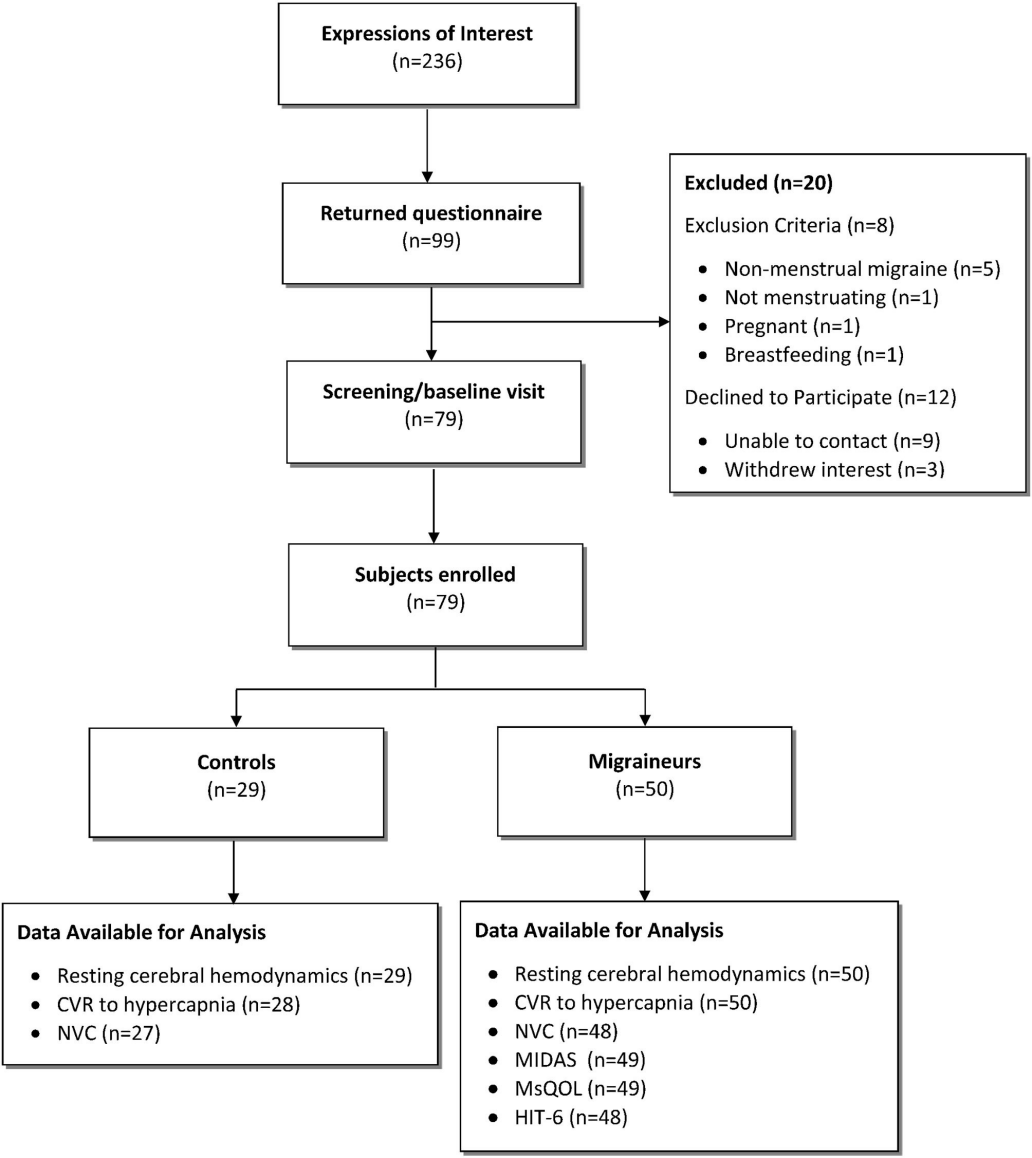
CONSORT diagram showing flow of study participants.

In total, 79 participants were enrolled into the study: 50 hormonal migraineurs, and 29 controls (Figure 1).

Participant characteristics are summarised in Table 1. There were no significant differences in age, systolic or diastolic blood pressure, small or large artery elasticity indexes or hormonal contraception use between hormonal migraineurs and controls. However, heart rate was significantly higher in hormonal migraineurs than controls. Additionally, controls tended to have a higher accuracy for the cognitive tests (1-Back, 2-Back 1.5 s and 2-Back 1.0 s) compared to hormonal migraineurs; this, however, did not reach statistical significance.

**Table 1:** Participant Characteristics for Controls and Hormonal Migraineurs.

**Variables**	**Controls (*N* = 29)**	**Hormonal migraineurs (*N* = 50)**	***P***
Age (years)	35.6 ± 1.8	38.7 ± 1.2	0.152
BMI (kg/m^2^)	24.8 ± 1.2	25.4 ± 0.6	0.304
Systolic BP (mmHg)^a^	114.1 ± 2.5	116.0 ± 1.6	0.504
Diastolic BP (mmHg)^a^	67.1 ± 1.6	70.5 ± 1.2	0.095
HR (bpm)^a^	66.9 ± 1.4	73.9 ± 1.6	**0.004**
SAEI (ml/mmHg × 100)	8.0 ± 0.8	6.9 ± 0.4	0.525
LAEI (ml/mmHg × 10) ^a^	13.9 ± 0.7	13.4 ± 0.5	0.576
1-Back accuracy (%)	99.4 ± 0.6	97.5 ± 1.0	0.070
2-Back 1.5 s accuracy (%)	91.1 ± 1.6	88.1 ± 1.4	0.082
2-Back 1.0 s accuracy (%)	88.6 ± 1.4	84.6 ± 1.5	0.128
Hormonal contraception use (*N*)^b^	9	13	0.630
Right-handed dominance (*N*)^b^	27	46	0.640

*BMI, body mass index; BP, blood pressure; HR, heart rate; LAEI, large artery elasticity index; SAEI, small artery elasticity index; P, significance of difference between means*.

a *Student's t-test (all other continuous variables were evaluated using the Wilcoxon Mann-Whitney test)*.

b *Pearson Chi-Square test. Data missing for SAEI (N = 2), LAEI (N = 2), 1-Back Accuracy (N = 2), 2-Back 1.5 s Accuracy (N = 1) and 2-Back 1.0 s Accuracy (N = 1). P-values in bold indicate a statistically significant difference (P < 0.05) between the two groups*.

### Cerebrovascular Function

#### Resting Cerebral Haemodynamics

**Figure 2:**
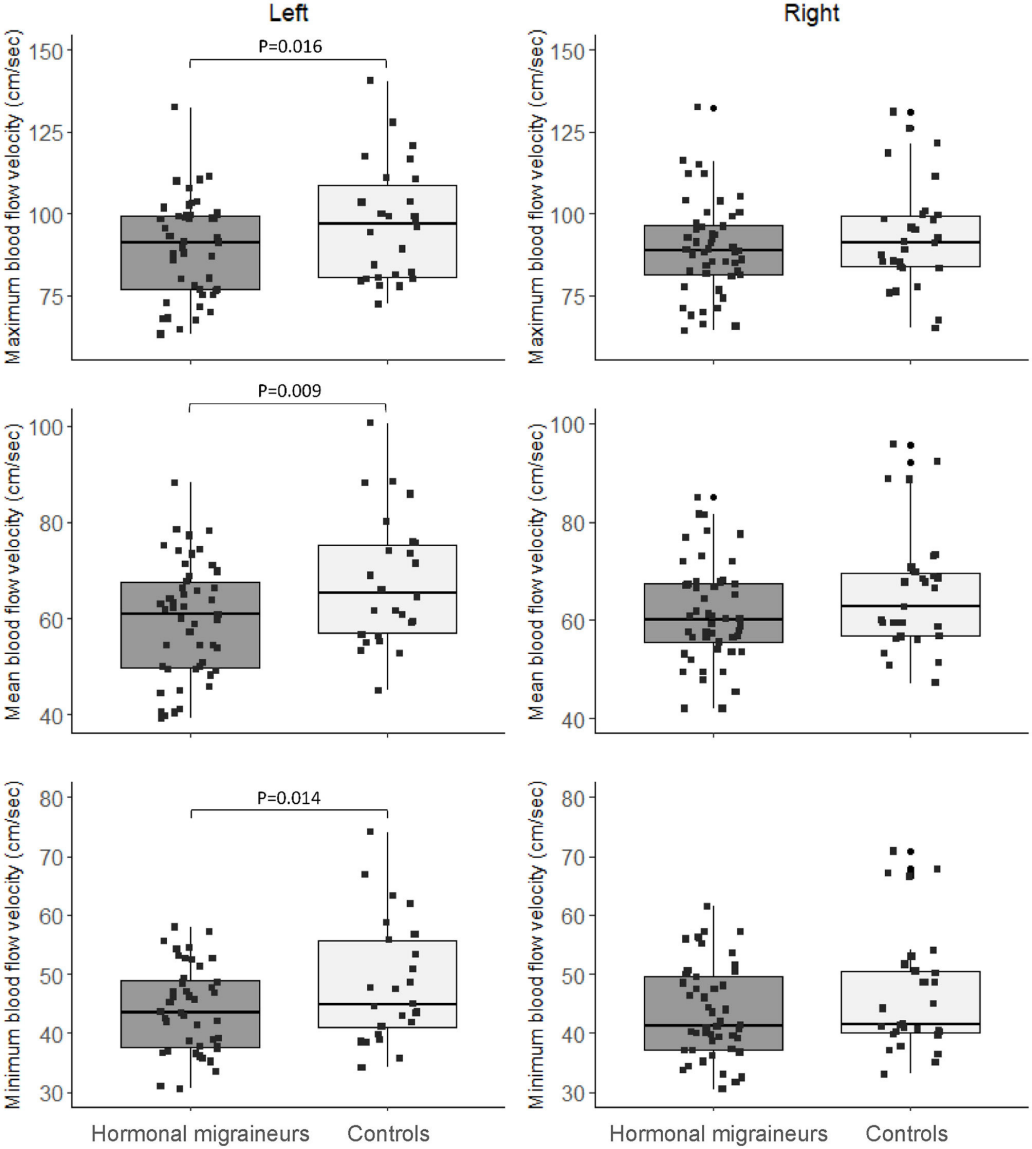
Box-and-whisker plot and scatter plot of resting maximum, mean and minimum blood flow velocity in the left and right MCA.

Hormonal migraineurs had significantly lower maximum, mean and minimum resting cerebral blood flow velocities than controls in the left MCA only (Figure 2). There were no significant bilateral differences in PI and RI between hormonal migraineurs and controls (Table 2).

**Table 2:** Cerebrovascular function in the middle cerebral artery of controls and hormonal migraineurs.

**Variables**	**Controls (*N* = 29)**	**Hormonal migraineurs (*N* = 50)**	***P***
Resting cerebral haemodynamics(cm/s)			
Systolic BFV (L)	97.0 ± 3.5	86.5 ± 2.5	**0.016**
Systolic BFV (R)	65.7 ±2.3	61.0 ± 1.5	0.155
Mean BFV (L)	67.7 ± 2.6	59.6 ± 1.7	**0.009**
Mean BFV (R)^a^	65.7 ± 2.3	61.0 ±1.5	0.132
Diastolic BFV (L)	47.7 ± 2.1	41.9 ± 1.2	**0.014**
Diastolic BFV (R)^a^	46.6 ± 1.9	42.9 ±1.2	0.146
PI (L)^a^	0.73 ± 0.03	0.74 ±0.02	0.697
PI (R)^a^	0.71 ± 0.03	0.74 ± 0.02	0.424
RI (L)^a^	0.50 ± 0.01	0.50 ± 0.01	0.652
RI (R)	0.50 ± 0.01	0.50 ± 0.01	0.569
CVR to hypercapnia (%)			
CVR to hypercapnia (L)^a^	32.9 ± 2.0	40.3 ± 2.7	0.171
CVR to hypercapnia (R)	34.2 ± 2.3	39.5 ± 2.3	0.140
Neurovascular coupling (%)			
1-Back (L)	12.7 ± 2.2	11.9 ± 1.1	0.730
1-Back (R)	11.9 ± 1.7	11.8 ± 1.2	0.955
2-Back 1.5 s (L)^a^	15.2 ± 2.2	13.5 ± 1.1	0.444
2-Back 1.5 s (R)	15.5 ± 2.0	15.0 ± 1.1	0.819
2-Back 1.0 s (L)	17.1 ± 2.0	10.3 ± 1.5	**0.010**
2-Back 1.0 s (R)	15.1 ± 1.8	12.1 ± 1.6	0.231

*BFV, blood flow velocity; CVR, cerebrovascular responsiveness; L, left side; R, right side; P, significance of difference between means; PI, pulsatility index; RI, resistive index. Data missing for resting cerebral haemodynamics (n = 3), CVR to hypercapnia (n = 2), 1-Back (n = 4) and 2-Back (n = 5) in left MCA; CVR to hypercapnia (n = 1), 1-Back (n = 3), and 2-Back (n = 2) in right MCA for controls. Data missing for 1-Back (n = 1), 2-Back 1.5 s (n = 3,) and 2-Back 1.0 s (n = 2) in left MCA; 1-Back (n = 2) and 2-Back (n = 3) in right MCA for hormonal migraineurs*.

a *Wilcoxon Mann-Whitney test (all other continuous variables were evaluated using the Student's t-test). P-values in bold indicate a statistically significant difference (P < 0.05) between the two groups*.

#### CVR to Hypercapnia

Hormonal migraineurs and controls were found to have similar CVR to hypercapnia in both left (controls 32.9 ± 2.0 %, hormonal migraineurs 40.3 ± 2.7 %; Cohen's *d* = 0.479; *P* = 0.171; Wilcoxon-Mann-Whitney test) and right MCA (controls 34.2 ± 2.3 %, hormonal migraineurs 39.5 ± 2.3 %; Cohen's *d* = 0.367; *P* = 0.140; *t*-test).

#### Neurovascular Coupling

**Figure 3:**
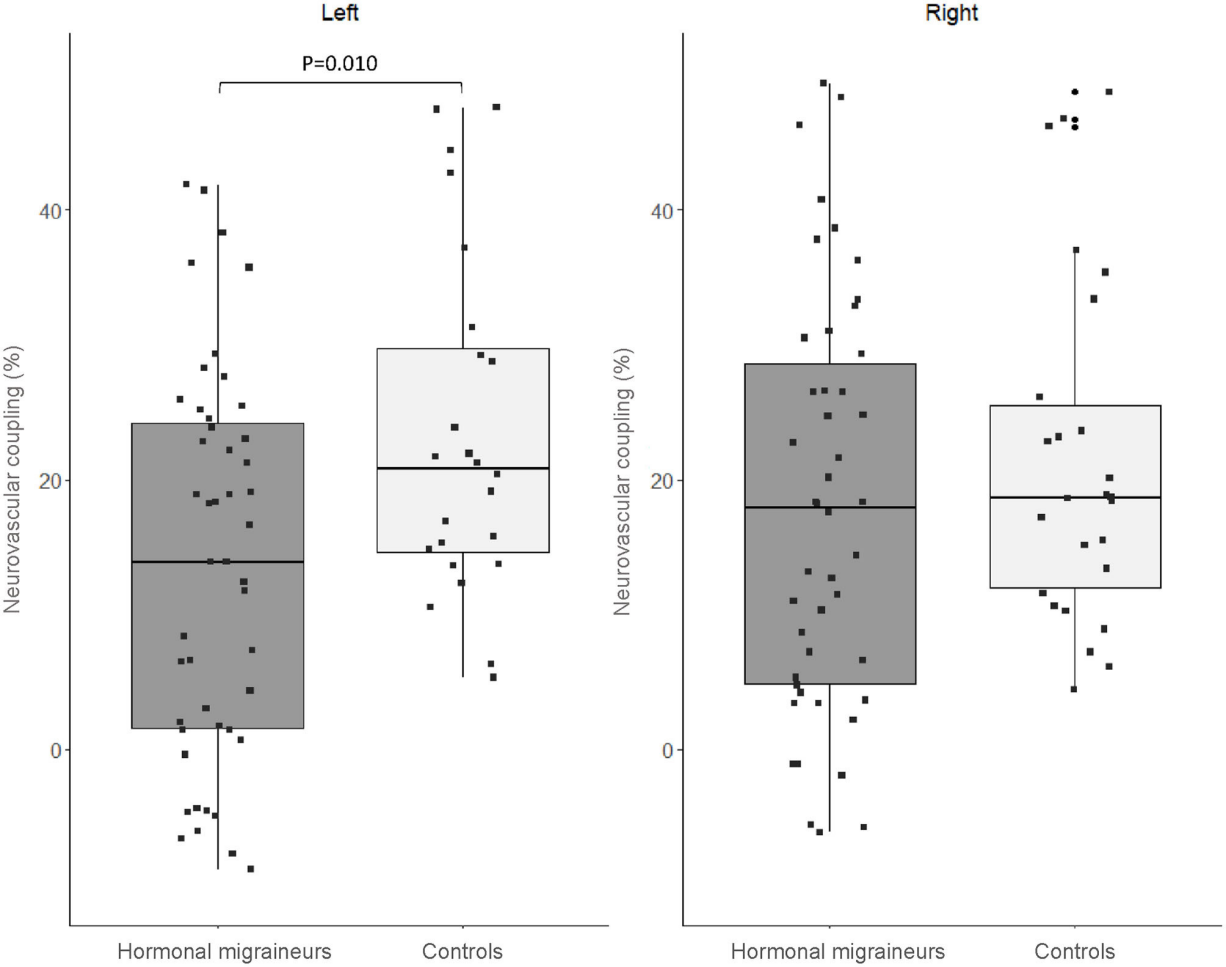
Box-and-whisker plot and scatter plot of neurovascular coupling during the N-Back test (2-Back 1.0 s) in the left and right MCA of migraineurs and controls.

There were no significant differences in neurovascular coupling during the 1-Back test and the 2-Back 1.5 s test between hormonal migraineurs, and controls in either the left or right MCA. However, in the left MCA, neurovascular coupling during the 2-Back 1.0 s test was 39.6% lower (*P* = 0.010) in hormonal migraineurs than controls (Figure 3). A paired *t*-test revealed a significant difference (*P* = 0.048) in neurovascular coupling between the left and right MCA during the 2-Back 1.0 s test in the hormonal migraine cohort (Table 3).

**Table 3:** A comparison of cerebrovascular function in the left and right middle cerebral artery of controls and hormonal migraineurs.

**Variables (left MCA vs. right MCA)**	***P***	
	**Controls (*N* = 29)**	**Hormonal migraineurs (*N* = 50)**
Resting cerebral haemodynamics (cm/s)		
Systolic BFV	0.440	0.409
Mean BFV	0.487	0.374
Diastolic BFV	0.538	0.391
PI^a^	0.547	0.898
RI^a^	0.612	0.888
CVR to hypercapnia (%)		
CVR to hypercapnia^a^	0.101	0.754
Neurovascular coupling (%)		
1-Back	0.876	0.933
2-Back 1.5 s^a^	0.128	0.081
2-Back 1.0 s	0.339	**0.048**

*BFV, blood flow velocity; CVR, cerebrovascular responsiveness; L, left side; R, right side; P, significance of difference between means*.

a *Wilcoxon Signed Rank test (all other continuous variables were evaluated using the paired samples t-test). P-values in bold indicate a statistically significant difference (P < 0.05) between the left and right middle cerebral artery*.

### Migraine-Related Disability, Quality of Life and Migraine Characteristics

#### Migraine-Related Disability and Quality of Life

The scores for the MIDAS, HIT-6™ and MSQoL migraine questionnaires are summarised in Table 4. Hormonal migraineurs scored 64.9 ± 1.0 and 22.7 ± 3.5 for the HIT-6™ and MIDAS questionnaires, respectively, which placed them in the severe grade for headache impact and migraine-related disability. The MSQoL scores for the role-function restrictive, role-function preventive and emotional function domains indicated that migraine was having a severe impact on quality of life, with a lower score reflecting a more severe impact on quality of life. The degree to which migraine limited our hormonal migraine cohort's ability to undertake daily activities (role-function restrictive domain) was the most affected whereas the degree to which migraine prevented our cohort's ability to undertake daily activities (role-function preventive domain) was the least impacted.

**Table 4:** Migraine-related disability and quality of life in hormonal migraineurs.

**Questionnaire**	**Mean score ± SEM**
HIT-6™ (n = 48)	64.9 ± 1.0
MIDAS (n = 49)	22.7 ± 3.5
MIDAS item A (headache frequency)	10.3 ± 1.2
MIDAS item B (headache severity)	7.3 ± 0.3
Migraine headache days (monthly)	3.4 ± 0.4
MSQoL (n = 49)^a^	
Role function restrictive	36.0 ± 3.6
Role function preventive	52.6 ± 3.9
Emotional function	37.6 ± 4.2

*The HIT-6™ is scored between 36 and 78; the higher the score, the higher the impact of migraine on an individual's ability to function at work, home and during social situations. The MIDAS is scored between 0 and 270; the higher the MIDAS score, the higher the migraine-related disability. MIDAS item A assesses headache frequency during the last three previous months at the time of the clinic visit. MIDAS item B assesses the average intensity of headache pain during the last three previous months at the time of the clinic visit using an 11-point (0–10) numeric pain rating scale. The higher the MSQoL, the better the self-perceived quality of life. Data missing for HIT-6 (n = 2), MIDAS (n = 1) and MSQoL (n = 1). MIDAS, Migraine Disability Assessment; HIT-6™, Headache Impact Test-6; MSQoL, Migraine-Specific Quality of Life*.

a *Scaled score from 0 to 100 (all other questionnaire data is presented as the raw score)*.

#### Migraine Characteristics (Frequency and Intensity) as Assessed by the MIDAS

The mean frequency and intensity of migraine experienced by the hormonal migraine group during the last three previous months at the time of the clinic visit is summarised in Table 4. During the last three previous months, the hormonal migraineurs experienced 10.3 ± 1.2 days with hormonal migraine. Additionally, hormonal migraineurs scored 7.3 ± 0.3 on the 11-point (0–10) numeric pain rating scale, reflecting moderate to severe migraine pain intensity.

### Univariate and Multi-Variate Linear Regression Analyses

Supplementary Tables 1, 2 show predictors of cerebrovascular function (resting MBFV, CVR to hypercapnia, and neurovascular coupling) in the total study population. Univariate analysis revealed age and SBP to be predictors of resting MBFV in the left MCA, and HR, SBP, and DBP to be predictors of neurovascular coupling (2-Back 1.0 s) in the left MCA. After multi-variate analysis was performed, age was the only predictor that maintained its significant association.

#### Associations Between Cerebrovascular Function and Migraine Related Quality of Life, Disability and Migraine Characteristics (Frequency and Intensity)

Univariate analyses revealed a significant negative correlation between headache frequency and CVR to hypercapnia in the left (*P* = 0.026; *r*^2^ = 0.101) and right MCA (*P* = 0.044; *r*^2^ = 0.083); this association was only maintained in the left MCA after multi-variate analysis was performed. Additionally, there was a significant negative correlation between neurovascular coupling during the 2-Back 1.5 s task in the right MCA and the MSQoL emotional (*P* = 0.013; *r*^2^ = 0.132), and role-function restrictive domains (*P* = 0.039; *r*^2^ = 0.094); these associations were not maintained after multi-variate analysis was performed. An association between MIDAS and neurovascular coupling during the 2-back 1.0 s task in the left MCA was found (*P* = 0.059; *r*^2^ = 0.077); however this significant association was not maintained in multi-variate analysis. No other significant associations between cerebrovascular function, migraine-related quality of life and disability or headache frequency, and intensity were found (Supplementary Tables 3, 4).

## Discussion

### Cerebrovascular Function

To our knowledge, this is the first study to show that hormonal migraineurs have poorer cerebrovascular function than controls when free from migraine and not menstruating, as demonstrated by impaired neurovascular coupling, particularly during the more complex cognitive tests (23, 24). Neurovascular coupling is where transient increases in neuronal activity are matched with local increases in cerebral perfusion (25, 26). Thus, impaired neurovascular coupling may result in inadequate perfusion of active brain regions, consistent with a tendency to poorer cognitive performance in the migraineurs. Hu et al. found migraineurs to have lower neurovascular coupling during a cognitive test battery in the left angular, parietal and marginal gyri but higher neurovascular coupling in the right occipital and parietal gyri compared to controls, as measured using magnetic resonance imaging (MRI). Their finding of lower neurovascular coupling in the left hemisphere of migraineurs is in agreement with our finding of lower neurovascular coupling in the left MCA during the most challenging N-Back test (2-Back 1.0 s). The reduced neurovascular coupling in the left hemisphere might be attributed to repetitive nociceptive stimulation whilst the higher neurovascular coupling in the right hemisphere might be compensatory. Furthermore, 92% of their migraine cohort suffered from left-sided or bilateral migraine (24), suggesting that the predominant migraine side may be associated with poorer cerebrovascular function. We could not confirm this hypothesis in this study, as we did not collect data on migraine pain laterality, which is a limitation of our study. Nonetheless, we found a significant difference between the left and right neurovascular coupling in the MCA during the 2-Back 1.0 s test in hormonal migraineurs only; neurovascular coupling in the left MCA was significantly lower than the right MCA (27).

Another possible explanation for the lateralisation of impaired neurovascular coupling to the left hemisphere or side involves sub-vocal rehearsal (i.e., the rehearsal of presented material, such as words, letters or sounds using the “inner voice”) (28). Sub-vocal rehearsal has been shown to activate brain regions of the left hemisphere and is believed to be involved in verbal working memory (29–31). The N-Back tests using letters have been shown to activate the frontotemporal region in the left hemisphere (32). In this study, the N-Back test can be considered a verbal working memory test as the letters presented in the test can be rehearsed by the inner voice (32, 33). Although the hormonal migraineurs in this study tended to perform poorer than controls in both conditions of the 2-Back (1.5 and 1.0 s), their cognitive performance did not correlate with neurovascular coupling during the N-Back. This was as expected in a young and cognitively unimpaired population.

Few studies have compared resting cerebrovascular function between hormonal migraineurs and healthy controls. We found hormonal migraineurs to have poorer cerebrovascular function as represented by lower resting MBFV in the left MCA compared to controls (27). However, in a small sample of five hormonal migraineurs and five controls, Ances and Detre (34) reported no difference in resting CBF in the occipital lobe. Our finding of lower resting MBFV in hormonal migraineurs conflicted with our recently published meta-analysis (12). The review found migraineurs have higher resting MBFV in the blood vessels that form the anterior cerebral circulation (viz. anterior cerebral artery, middle cerebral artery). However, none of the studies included in the meta-analysis for MBFV in the anterior cerebral circulation were conducted in hormonal migraineurs, suggesting that resting cerebrovascular function in the latter may differ from that of non-hormonal migraineurs (12).

We found no significant difference in PI or CVR to hypercapnia between hormonal migraineurs and controls. Tasdemir et al. (35) also found no difference in PI between 23 hormonal migraineurs and 20 controls. Interestingly, they found CVR to hypercapnia in both the MCA and the posterior cerebral artery to be lower in hormonal migraineurs. We did not assess CVR to hypercapnia in the posterior circulation, which is a limitation of our study as our findings may not be applicable to other cerebral arteries, particularly those that form the posterior cerebral circulation (viz. basilar artery, posterior cerebral artery, vertebral artery). Nonetheless, the findings by Tasdemir et al. (35) may have been limited by their methodology; cerebrovascular function was measured on day 3 and 10 of the menstrual cycle in their hormonal migraineurs and on any day of the cycle in controls. Cerebrovascular function has been reported to vary considerably throughout the menstrual cycle (36, 37) as further demonstrated by their findings of significantly increased CVR to hypercapnia on the 10th day compared to the third day of the menstrual cycle (35). Additionally, cerebrovascular function has been shown to differ between different phases of the menstrual cycle with Brackley et al. (37) reporting higher intracranial stiffness in the MCA of healthy women during the luteal phase compared to the follicular phase.

Differences in CVR have been reported to be more readily elicited by cognitive stimuli than ventilatory stimuli (38). This may explain why we did not see any difference in CVR to hypercapnia between hormonal migraineurs and controls (38).

Nonetheless, it would be prudent to see whether other measures of cerebrovascular function such as dynamic or static cerebral autoregulation are associated with hormonal migraine. In this present study, SBP and/or DBP were identified as predictors for MBFV, CVR to hypercapnia, and neurovascular coupling. Currently, it is unclear whether migraine is associated with altered cerebral autoregulation (12). Additionally, to our knowledge, no previous study has compared cerebral autoregulation in hormonal migraineurs with healthy controls.

Together with poorer cerebrovascular function, our hormonal migraineurs had a higher resting HR than controls. This observation is consistent with previous studies showing an association between high resting HR and increased risk of cardiovascular and cerebrovascular disease in the general population (39) and warrants further investigation.

### Migraine-Related Disability and Quality of Life

Our HIT-6™ and MIDAS scores revealed that migraine was having a severe impact on quality of life and causing severe disability in our hormonal migraineurs. Our HIT-6™ and MIDAS scores were, for the most part, comparable to other studies that assessed these questionnaires in hormonal migraineurs (40, 41). However, to our knowledge, no previous study has assessed migraine-related disability in a population of hormonal migraineurs using the MSQoL. Nonetheless, Young et al. (42) found chronic migraineurs who suffered from daily headaches had slightly higher baseline MSQoL scores than our cohort.

The minimal important difference (MID) measures the smallest detectable difference in HIT-6™, MIDAS and MSQoL questionnaire scores between two groups that migraineurs recognise as important (i.e., reflecting worsening or improvement of their condition) (43–45). In this study, the MID for the HIT-6™ and MIDAS questionnaires were −3.5 points and −12.1 points (46), which was greater than the −1.5 points outlined by Smelt et al. (44) for the HIT-6™ and the −5 points set by Lipton et al. (43) for the MIDAS questionnaire. This indicated that our cohort require a greater decrease in points for the MIDAS and HIT-6™ questionnaires to reflect an improvement in quality of life.

We were unable to detect meaningful associations between migraine-related disability or quality of life and cerebrovascular function. Several factors may have limited our ability to detect meaningful associations between these outcomes; this will be discussed in the limitations.

### Limitations

Whilst this study has identified some important differences in cerebrovascular function between hormonal migraineurs and controls, it is only a small exploratory study; a larger study with an adjustment for multiple comparisons may have detected significant differences in other parameters. Secondly, we may not have been able to detect any meaningful associations between migraine-related disability or quality of life measures and cerebrovascular function as the recall period for the MIDAS, HIT-6 and MSQoL questionnaires range from 4 weeks to 3 months whereas cerebrovascular function was only assessed at one time point. Thirdly, whilst all participants visited the research centre when they were not menstruating and free from migraine, we did not control for the menstrual cycle phase (i.e., follicular, luteal). Therefore, hormonal migraineurs and controls may have visited the research centre for testing during different phases of the menstrual cycle; this may have limited the comparability between the two groups (37, 47). Fourthly, we did not exclude participants who were taking hormonal or migraine medications. Previous studies have shown that both hormonal or migraine medications may affect cerebrovascular function (48, 49); thus, this may have limited our findings. Lastly, we used a slightly modified version of the International Classification of Headache Disorders (ICHD-III) criteria for the recruitment of hormonal migraine participants (8).

## Conclusions and Future Directions

In summary, this one-time point cross-sectional study has shown that hormonal migraineurs have poorer cerebrovascular function than controls as represented by lower resting MBFV and neurovascular coupling in the left MCA. The hormonal migraineurs also had a higher resting HR than controls. These observations, implicate abnormal cardiovascular and, in particular, cerebrovascular function in the pathogenesis of hormonal migraine. Due to the cross-sectional nature of our study and data collection only from a single point in time, we are unable to prove a causal relationship between poor cerebrovascular function and hormonal migraine. Therefore, future prospective longitudinal studies are needed to validate our findings. In addition, future studies should also include a non-hormonal migraine control group to ascertain whether hormonal migraineurs, non-hormonal migraineurs and non-migraineurs have differing cerebrovascular function. Our migraine-related disability and quality of life measures were for the most part comparable with other studies conducted in hormonal migraineurs; the HIT-6™ and MIDAS questionnaires showed that migraine was having a severe impact on our migraine cohorts' quality of life. Future intervention studies should utilise the MID to investigate whether improvements in cerebrovascular function are associated with improvements in migraine-related quality of life, disability, frequency and intensity.

## Data Availability Statement

The original contributions presented in the study are included in the article/Supplementary Material, further inquiries can be directed to the corresponding author.

## Ethics Statement

The studies involving human participants were reviewed and approved by The University of Newcastle Human Research Ethics Committee. The patients/participants provided their written informed consent to participate in this study.

## Author Contributions

RW and PH conceived and designed the study. JD collected and analysed the data, and interpreted the results under the supervision of RW and PH. JD drafted the manuscript under the guidance of RW, PH, LG, and KC. All authors contributed to the article and approved the submitted version.

## Conflict of Interest

The authors declare that the research was conducted in the absence of any commercial or financial relationships that could be construed as a potential conflict of interest.

## Abbreviations

ACTRN: Australian Clinical Trials Registry Number
BFV: Blood flow velocity
CBF: Cerebral blood flow
CVR: Cerebrovascular responsiveness
HIT-6™: Headache Impact Test-6
HR: Heart rate
MBFV: Mean blood flow velocity
MID: Minimal important difference
MCA: Middle cerebral artery
MIDAS: Migraine Disability Assessment
MSQoL: Migraine-Specific Quality of Life
PI: Pulsatility index
RI: Resistive index
TCD: Transcranial Doppler.
