# Supplementary material for: Cerebrovascular Function in Hormonal Migraine: An Exploratory Study
Source: Front Neurol. 2021 Jul 7;12:694980. doi: 10.3389/fneur.2021.694980 (PMC8292610; doi:10.3389/fneur.2021.694980)
Supplement: Supplementary file 1 [file Table_1.docx]

**Supplemental Table 1**: **Results from Univariate and Multivariate Regression Model in the Total Study Population for MBFV and CVR to Hypercapnia in the Left and Right MCA.** Abbreviations: β, standardised beta coefficient; BMI, body mass index; DBP, diastolic blood pressure; HR, heart rate; MBFV, mean blood flow velocity; MCA, middle cerebral artery. p<0.1 considered significant for univariate analyses; p<0.05 considered significant for multivariate analyses. *DBP not used in multivariate analysis due to collinearity with SBP.

| **Total Population** | **Left MCA** | | | | **Right MCA** | | | |
| --- | --- | --- | --- | --- | --- | --- | --- | --- |
|  | **Univariate** | | **Multivariate** | | **Univariate** | | **Multivariate** | |
| **MBFV** | **β** | **P** | **β** | **P** | **β** | **P** | **β** | **P** |
| **Age** | -0.329 | **0.004** | -0.296 | **0.012** | -0.179 | 0.114 | - | - |
| **BMI** | 0.062 | 0.593 | - | - | 0.083 | 0.468 | - | - |
| **HR** | 0.035 | 0.767 | - | - | 0.059 | 0.607 | - | - |
| **SBP** | -0.197 | **0.087** | -0.107 | 0.359 | -0.125 | 0.273 | - | - |
| **DBP** | -0.169 | 0.145 | - | - | -0.090 | 0.428 | - | - |
|  | **Univariate** | | **Multivariate** | | **Univariate** | | **Multivariate** | |
| **CVR to Hypercapnia** | **β** | **P** | **β** | **P** | **β** | **P** | **β** | **P** |
| **Age** | 0.010 | 0.934 | - | - | 0.080 | 0.487 | - | - |
| **BMI** | -0.093 | 0.425 | - | - | -0.105 | 0.360 | - | - |
| **HR** | 0.266 | **0.020** | 0.220 | 0.073 | 0.206 | **0.071** | 0.149 | 0.209 |
| **SBP** | 0.155 | 0.182 | - | - | 0.225 | **0.047** | 0.178 | 0.134 |
| **DBP** | 0.206 | **0.075** | 0.123 | 0.312 | 0.254 | **0.025*** | - | - |
