# Supplementary material for: Cerebrovascular Function in Hormonal Migraine: An Exploratory Study
Source: Front Neurol. 2021 Jul 7;12:694980. doi: 10.3389/fneur.2021.694980 (PMC8292610; doi:10.3389/fneur.2021.694980)
Supplement: Supplementary file 2 [file Table_2.docx]

**Supplemental Table 2**: **Results from Univariate and Multivariate Regression Model in the Total Study Population for Neurovascular Coupling in the Left and Right MCA.** Abbreviations: β, standardised beta coefficient; BMI, body mass index; DBP, diastolic blood pressure; HR, heart rate; MBFV, mean blood flow velocity; MCA, middle cerebral artery p<0.1 considered significant for univariate analyses; p<0.05 considered significant for multivariate analyses. *DBP not used in multivariate analysis due to collinearity with SBP.

| **Total Population** | **Left MCA** | | | | **Right MCA** | | | |
| --- | --- | --- | --- | --- | --- | --- | --- | --- |
|  | **Univariate** | | **Multivariate** | | **Univariate** | | **Multivariate** | |
| **1-Back** | **β** | **P** | **β** | **P** | **β** | **P** | **β** | **P** |
| **Age** | 0.004 | 0.973 | - | - | 0.123 | 0.292 | - | - |
| **BMI** | -0.160 | 0.173 | - | - | 0.019 | 0.870 | - | - |
| **HR** | -0.139 | 0.236 | - | - | -0.028 | 0.813 | - | - |
| **SBP** | -0.261 | **0.025** | - | - | -0.140 | 0.231 | - | - |
| **DBP** | -0.210 | **0.073*** | - | - | -0.118 | 0.315 | - | - |
|  | **Univariate** | | **Multivariate** | | **Univariate** | | **Multivariate** | |
| **2-Back 1.5s** | **β** | **P** | **β** | **P** | **β** | **P** | **β** | **P** |
| **Age** | -0.083 | 0.492 | - | - | 0.062 | 0.604 | - | - |
| **BMI** | -0.107 | 0.374 | - | - | -0.056 | 0.641 | - | - |
| **HR** | 0.003 | 0.981 | - | - | 0.195 | **0.098** | - | - |
| **SBP** | -0.208 | **0.082** | - | - | -0.001 | 0.990 | - | - |
| **DBP** | -0.190 | 0.113 | - | - | 0.002 | 0.987 | - | - |
|  | **Univariate** | | **Multivariate** | | **Univariate** | | **Multivariate** | |
| **2-Back 1.0s** | **β** | **P** | **β** | **P** | **β** | **P** | **β** | **P** |
| **Age** | -0.172 | 0.149 | - | - | -0.138 | 0.245 | - | - |
| **BMI** | -0.042 | 0.727 | - | - | 0.008 | 0.948 | - | - |
| **HR** | -0.201 | **0.090** | -0.131 | 0.296 | -0.204 | **0.084** | -0.142 | 0.245 |
| **SBP** | -0.243 | **0.039** | -0.196 | 0.118 | -0.239 | **0.042** | -0.193 | 0.115 |
| **DBP** | -0.260 | **0.027*** | - | - | -0.246 | **0.036*** | - | - |
