# Supplementary material for: Cerebrovascular Function in Hormonal Migraine: An Exploratory Study
Source: Front Neurol. 2021 Jul 7;12:694980. doi: 10.3389/fneur.2021.694980 (PMC8292610; doi:10.3389/fneur.2021.694980)
Supplement: Supplementary file 3 [file Table_3.docx]

**Supplemental Table 3**: **Results from Univariate and Multivariate Regression Model in Hormonal Migraineurs Only for MBFV and CVR to Hypercapnia in the Left and Right MCA.** Abbreviations:β, standardised beta coefficient; BMI, body mass index; DBP, diastolic blood pressure; EF, emotional function domain; HR, heart rate; HIT-6; headache impact test -6; MBFV, mean blood flow velocity; MCA, middle cerebral artery; MIDAS, migraine disability assessment; RFR, role-function restrictive domain; RFP, role-function preventive domain. p<0.1 considered significant for univariate analyses; p<0.05 considered significant for multivariate analyses. *DBP not used in multivariate analysis due to collinearity with SBP.

| **Hormonal Migraineurs** | **Left MCA** | | | | **Right MCA** | | | | |
| --- | --- | --- | --- | --- | --- | --- | --- | --- | --- |
|  | **Univariate** | | **Multivariate** | | **Univariate** | | **Multivariate** | | |
| **MBFV** | **β** | **P** | **β** | **P** | **β** | **P** | **β** | **P** |  |
| **Age** | -0.377 | **0.007** | - | **-** | -0.194 | 0.176 | - | - |  |
| **BMI** | 0.029 | 0.843 | - | - | 0.022 | 0.881 | - | - |  |
| **HR** | 0.013 | 0.929 | - | - | 0.013 | 0.927 | - | - |  |
| **SBP** | -0.223 | 0.119 | - | - | -0.139 | 0.335 | - | - |  |
| **DBP** | -0.155 | 0.284 | - | - | -0.102 | 0.483 | - | - |  |
| **MIDAS** | -0.150 | 0.303 | - | - | 0.050 | 0.731 | - | - |  |
| **Headache frequency** | -0.109 | 0.749 | - | - | 0.109 | 0.455 | - | - |  |
| **Headache severity** | -0.205 | 0.158 | - | - | -0.025 | 0.865 | - | - |  |
| **HIT-6** | 0.028 | 0.848 | - | - | 0.161 | 0.274 | - | - |  |
| **RFR** | -0.029 | 0.841 | - | - | -0.211 | 0.145 | - | - |  |
| **RFP** | 0.033 | 0.820 | - | - | -0.216 | 0.136 | - | - |  |
| **EF** | 0.141 | 0.333 | - | - | -0.068 | 0.642 | - | - |  |
|  | **Univariate** | | **Multivariate** | | **Univariate** | | **Multivariate** | | |
| **CVR to Hypercapnia** | **β** | **P** | **β** | **P** | **β** | **P** | **β** | **P** |  |
| **Age** | -0.026 | 0.858 | - | - | 0.082 | 0.569 | - | - |  |
| **BMI** | -0.002 | 0.989 | - | - | -0.082 | 0.571 | - | - |  |
| **HR** | 0.282 | **0.047** | -0.273 | 0.063 | 0.277 | **0.051** | 0.225 | 0.119 |  |
| **SBP** | 0.293 | **0.039** | 0.133 | 0.363 | 0.378 | **0.007** | 0.252 | 0.086 |  |
| **DBP** | 0.309 | **0.029*** | - | - | 0.389 | **0.005*** | - | - |  |
| **MIDAS** | -0.101 | 0.489 | - | - | -0.020 | 0.891 | - | - |  |
| **Headache frequency** | -0.318 | **0.026** | -0.323 | **0.023** | -0.288 | **0.044** | -0.264 | 0.058 |  |
| **Headache severity** | -0.094 | 0.522 | - | - | 0.020 | 0.893 | - | - |  |
| **HIT-6** | -0.094 | 0.524 | - | - | -0.049 | 0.742 | - | - |  |
| **RFR** | 0.214 | 0.139 | - | - | 0.105 | 0.474 | - | - |  |
| **RFP** | 0.090 | 0.540 | - | - | -0.007 | 0.962 | - | - |  |
| **EF** | 0.025 | 0.865 | - | - | -0.082 | 0.574 | - | - |  |
