# Supplementary material for: Cerebrovascular Function in Hormonal Migraine: An Exploratory Study
Source: Front Neurol. 2021 Jul 7;12:694980. doi: 10.3389/fneur.2021.694980 (PMC8292610; doi:10.3389/fneur.2021.694980)
Supplement: Supplementary file 4 [file Table_4.docx]

**Supplemental Table 4**: **Results from Univariate and Multivariate Regression Model in Hormonal Migraineurs Only for Neurovascular Coupling in the Left and Right MCA.** Abbreviations: β, standardised beta coefficient; BMI, body mass index; DBP, diastolic blood pressure; EF, emotional function domain; HR, heart rate; HIT-6; headache impact test -6; MBFV, mean blood flow velocity; MCA, middle cerebral artery; MIDAS, migraine disability assessment; RFR, role-function restrictive domain; RFP, role-function preventive domain. p<0.1 considered significant for univariate analyses; p<0.05 considered significant for multivariate analyses.

| **Hormonal Migraineurs** | **Left MCA** | | | | **Right MCA** | | | |
| --- | --- | --- | --- | --- | --- | --- | --- | --- |
|  | **Univariate** | | **Multivariate** | | **Univariate** | | **Multivariate** | |
| **1-Back** | **β** | **P** | **β** | **P** | **β** | **P** | **β** | **P** |
| **Age** | 0.076 | 0.606 | - | - | 0.127 | 0.353 | - | - |
| **BMI** | 0.128 | 0.380 | - | - | 0.202 | 0.169 | - | - |
| **HR** | -0.101 | 0.490 | - | - | -0.062 | 0.673 | - | - |
| **SBP** | -0.141 | 0.333 | - | - | -0.213 | 0.287 | - | - |
| **DBP** | -0.158 | 0.280 | - | - | -0.113 | 0.441 | - | - |
| **MIDAS** | -0.204 | 0.164 | - | - | -0.116 | 0.437 | - | - |
| **Headache frequency** | 0.107 | 0.471 | - | - | 0.105 | 0.484 | - | - |
| **Headache severity** | -0.005 | 0.974 | - | - | 0.077 | 0.606 | - | - |
| **HIT-6** | -0.126 | 0.399 | - | - | 0.128 | 0.398 | - | - |
| **RFR** | -0.043 | 0.770 | - | - | 0.001 | 0.995 | - | - |
| **RFP** | 0.130 | 0.378 | - | - | -0.038 | 0.799 | - | - |
| **EF** | -0.129 | 0.382 | - | - | -0.178 | 0.232 | - | - |
|  | **Univariate** | | **Multivariate** | | **Univariate** | | **Multivariate** | |
| **2-Back 1.5s** | **β** | **P** | **β** | **P** | **β** | **P** | **β** | **P** |
| **Age** | -0.101 | 0.638 | - | - | 0.205 | 0.166 | - | - |
| **BMI** | 0.101 | 0.499 | - | - | 0.076 | 0.612 | - | - |
| **HR** | 0.189 | 0.204 | - | - | 0.340 | **0.019** | 0.157 | 0.314 |
| **SBP** | -0.003 | 0.983 | - | - | 0.276 | **0.060** | 0.214 | 0.155 |
| **DBP** | -0.088 | 0.559 | - | - | 0.219 | 0.138 | - | - |
| **MIDAS** | -0.008 | 0.958 | - | - | 0.211 | 0.160 | - | - |
| **Headache frequency** | 0.211 | 0.160 | - | - | 0.089 | 0.555 | - | - |
| **Headache severity** | -0.078 | 0.608 | - | - | 0.033 | 0.826 | - | - |
| **HIT-6** | 0.095 | 0.536 | - | - | 0.175 | 0.249 | - | - |
| **RFR** | -0.184 | 0.220 | - | - | -0.306 | **0.039** | -0.100 | 0.626 |
| **RFP** | -0.048 | 0.753 | - | - | -0.230 | 0.124 | - | - |
| **EF** | -0.281 | **0.058** | - | - | -0.363 | **0.013** | -0.248 | 0.228 |
|  | **Univariate** | | **Multivariate** | | **Univariate** | | **Multivariate** | |
| **2-Back 1.0s** | **β** | **P** | **β** | **P** | **β** | **P** | **β** | **P** |
| **Age** | -0.164 | 0.266 | - | - | -0.135 | 0.365 | - | - |
| **BMI** | 0.108 | 0.465 | - | - | 0.216 | 0.144 | - | - |
| **HR** | -0.065 | 0.661 | - | - | -0.155 | 0.300 | - | - |
| **SBP** | -0.225 | 0.125 | - | - | -0.179 | 0.229 | - | - |
| **DBP** | -0.244 | **0.095** | -0.201 | 0.176 | -0.221 | 0.136 | - | - |
| **MIDAS** | -0.277 | **0.059** | -0.229 | 0.125 | -0.203 | 0.177 | - | - |
| **Headache frequency** | -0.080 | 0.593 | - | - | -0.178 | 0.236 | - | - |
| **Headache severity** | 0.010 | 0.948 | - | - | 0.038 | 0.802 | - | - |
| **HIT-6** | 0.015 | 0.921 | - | - | 0.086 | 0.576 | - | - |
| **RFR** | 0.042 | 0.782 | - | - | 0.039 | 0.795 | - | - |
| **RFP** | 0.073 | 0.626 | - | - | 0.049 | 0.747 | - | - |
| **EF** | -0.029 | 0.849 | - | - | -0.054 | 0.724 | - | - |
